# Attenuated Total Reflection-Fourier Transform Infrared (ATR-FTIR) Spectroscopy Analysis of Saliva for Breast Cancer Diagnosis

**DOI:** 10.1155/2020/4343590

**Published:** 2020-02-10

**Authors:** Izabella C. C. Ferreira, Emília M. G. Aguiar, Alinne T. F. Silva, Letícia L. D. Santos, Léia Cardoso-Sousa, Thaise G. Araújo, Donizeti W. Santos, Luiz R. Goulart, Robinson Sabino-Silva, Yara C. P. Maia

**Affiliations:** ^1^Laboratory of Nanobiotechnology, Institute of Biotechnology, Federal University of Uberlandia, Uberlandia 38405-302, Brazil; ^2^Department of Physiology, Institute of Biomedical Sciences, Federal University of Uberlandia, Uberlandia 38405-302, Brazil; ^3^Obstetric Division, University Hospital, Federal University of Uberlandia, Uberlandia 38405-320, Brazil; ^4^School of Medicine, Federal University of Uberlandia, Uberlandia 38405-320, Brazil

## Abstract

Saliva biomarkers using reagent-free biophotonic technology have not been investigated as a strategy for early detection of breast cancer (BC). The attenuated total reflection-Fourier transform infrared (ATR-FTIR) spectroscopy has been proposed as a promising tool for disease diagnosis. However, its utilization in cancer is still incipient, and currently saliva has not been used for BC screening. We have applied ATR-FTIR onto saliva from patients with breast cancer, benign breast disease, and healthy matched controls to investigate its potential use in BC diagnosis. Several salivary vibrational modes have been identified in original and second-derivative spectra. The absorbance levels at wavenumber 1041 cm^−1^ were significantly higher (*p* < 0.05) in saliva of breast cancer patients compared with those of benign patients, and the ROC curve analysis of this peak showed a reasonable accuracy to discriminate breast cancer from benign and control patients. The 1433–1302.9 cm^−1^ band area was significantly higher (*p* < 0.05) in saliva of breast cancer patients than in control and benign patients. This salivary ATR-FTIR spectral area was prevalidated as a potential diagnostic biomarker of BC. This spectral biomarker was able to discriminate human BC from controls with sensitivity and specificity of 90% and 80%, respectively. Besides, it was able to differentiate BC from benign disease with sensitivity and specificity of 90% and 70%, respectively. Briefly, for the first time, saliva analysis by ATR-FTIR spectroscopy has demonstrated the potential use of salivary spectral biomarkers (1041 cm^−1^ and 1433–1302.9 cm^−1^) as a novel alternative for noninvasive BC diagnosis, which could be used for screening purposes.

## 1. Introduction

Breast cancer is a complex and heterogeneous disease caused by several factors, and its dissemination involves a succession of clinical and pathological stages beginning with carcinoma in situ, progressing to invasive lesion and culminating in metastatic disease [1, 2]. According to the World Cancer Report 2014 from the World Health Organization (WHO), breast cancer was the type with the highest incidence and highest mortality in the female population worldwide (1.7 million) in both developing and developed countries [3]. Early diagnosis and proper treatment are the main advantages of breast cancer screening tests. Basically, breast cancer diagnostic comprises four conventional techniques: histopathology, mammography, ultrasonography, and magnetic resonance imaging (MRI). However, in general these techniques have critical limitations related to efficacy and production of false positive or false negative results [4, 5].

Therefore, the increasing worldwide incidence of breast cancer and the absence of sufficient reliable, cost-effective, and high-throughput methods for detection requires a search for other diagnostic tools. The attenuated total reflection-Fourier transform infrared (ATR-FTIR) spectroscopy is a fast, nondestructive, noninvasive, label- and reagent-free, inexpensive, sensitive, and highly reproducible physicochemical tool for characterization of biological molecules in fluids. FTIR requires only a small amount of sample for analysis with easy and quick preparation if necessary, and it allows automated and repetitive analyses, leading to nonsubjective evaluation of the sample [4, 6, 7]. Furthermore, ATR, the experimental configuration for FTIR spectra acquisition utilized in this study, presents high signal-to-noise ratio (SNR), does not present unwanted spectral contributions, and enables a sample to be analyzed without further preparation simply by placing it in direct contact with a crystal with a refractive index higher than the sample [8–11].

FTIR can effectively provide information concerning the structure and chemical composition of biological samples at the molecular level and then the characterization of proteins, lipids, nucleic acids, and carbohydrates. FTIR is also sensitive to detect changes in molecular compositions according to diseased state, providing fingerprints of biological samples, like tissues, cells, and biological fluids. The generation and progression of malignancy at the molecular level in cells occur before morphological alterations in cancer. FTIR spectroscopy is capable to show changes in carcinogenesis-related vibrational modes to several human cancers [8, 12–14]. Specifically for breast cancer, FTIR spectroscopy has been used for many purposes [15–24], mainly for detection [4, 25–28]. Most FTIR spectroscopy studies in breast cancer used normal breast tissue and breast tumors [4, 29–31], breast cell lines [11, 32, 33], and blood of breast cancer patients [25, 27]. To our knowledge, there are no studies using ATR-FTIR spectroscopy for breast cancer diagnosis using saliva as the biological sample.

Saliva is a complex and dynamic biological fluid composed of 98% water and 2% of other important compounds, such as electrolytes, mucus, enzymes, proteins/peptides, nucleic acids, and hormones. Most of the organic compounds of saliva are produced in the salivary glands; however, some molecules originated from a diseased process may be transported from the blood to acinar cells via transcellular or paracellular fluxes into the acinar lumen [34–36]. Then, salivary biomarkers can be exploited for the early diagnosis of some systemic diseases [36–39]. Among the advantages, saliva may reflect several physiological states of the body; is simple, fast and safe to collect; is convenient to store; is noninvasive and, compared to blood, is painless to the patient, and requires less handling during diagnostic proceeding [38, 40, 41].

Here, we tested the hypothesis that specific salivary vibrational modes can be used to discriminate patients with breast cancer from benign patients and matched healthy controls, which may prove that salivary spectral biomarkers are suitable in diagnosing breast cancer. In this manner, the aim of the present study was to establish specific salivary vibrational modes, analyzed by ATR-FTIR spectroscopy, to detect breast cancer fingerprints that are suitable for diagnosis.

## 2. Materials and Methods

### 2.1. Ethical Aspects and Study Subjects

The study was conducted at the Clinics' Hospital of the Federal University of Uberlandia (HC-UFU, Uberlandia, Minas Gerais, Brazil) under the approval of the UFU Research Ethics Committee (protocol number 064/2008) and based on the standards of the Declaration of Helsinki. All research were performed in accordance with the relevant guidelines and regulations. Written informed consent was obtained from all the participants of this study including controls and patients. The subjects were randomly selected from the population before performing routine breast cancer screening and/or surgery. Exclusion criteria were age below 18 years, primary tumor site other than the breast, and physical and/or mental inability to respond to the tools necessary for data collection. The study group included 30 subjects: 10 with confirmed breast cancer by clinical, histological, and pathologic examination; 10 with some benign breast disease, like fibroadenomas, atypical ductal hyperplasia, papilloma, or others; and 10 without pathological findings, the control group. In this study was used the tumor-node-metastasis (TNM) cancer classification, which is according to the American Joint Committee on Cancer (AJCC) and the International Union for Cancer Control (UICC). This classification evaluates the extent of the primary tumor (T), regional lymph nodes (N), and distant metastases (M) and provides staging based on T, N, and M [42].

### 2.2. Sample Collection and Preparation

For each participant, saliva samples were collected before surgery in Salivette® tubes (Sarstedt, Germany), consisting of a neutral cotton swab and a conical tube. The patient chewed the swab for three minutes, which was then returned to the tube that was covered with a lid. Then, the saliva from the swab was recovered by centrifugation for 2 minutes at 1000 ×*g* and stored at −20°C. Then, the saliva samples (200 *μ*L) were lyophilized overnight. This freeze-drying of the samples removes the strong water infrared light absorption from spectra which may mask the signal from the sample and may reduce the intensity of the compounds under investigation [25, 43].

### 2.3. ATR-FTIR Spectroscopy

The spectra were measured in the 4000 to 400 cm^−1^ wavenumber region using a FTIR spectrometer VERTEX 70/70v (Bruker Corporation, Germany) coupled with Platinum Diamond ATR, which consists of a diamond disc as an internal reflection element. The lyophilized sample was placed on the ATR crystal, and then the spectrum was recorded. The spectrum of air was used as a background before each sample analysis. Background and sample spectra were taken in a room with a temperature around 21–23°C, at a spectral resolution of 4 cm^−1^, and to each measurement 32 scans were performed.

### 2.4. Spectral Data Preprocessing

The original FTIR spectra were normalized, and the baseline was corrected using OPUS software. This software was also used to calculate absorbance of area under spectral regions that correspond to specific saliva components, applying parameters already described [43]. Second differentiation spectra from the original were carried out using the Savitzky–Golay method in Origin 9.1 software in order to accentuate the bands, resolve overlapped bands, and increase the accuracy of analysis by revealing the genuine biochemical characteristics [25, 44]. In the smoothing pretreatment, the parameters of the Savitzky–Golay filter such as the polynomial order and points of window were chosen in order to find the relatively optimum smoothing effect. The parameters were set as 2 for polynomial order and 20 for points of window examined. The second derivative gives negative peaks (valleys) instead of bands from the original absorption spectrum. Therefore, the analyzed wavenumbers in the second derivative are the height of valleys.

### 2.5. Statistical Analysis

After the spectral preprocessing, the original and derivative values were used on the statistical analysis. First, values of absorbance at specific wavenumbers and spectral regions were submitted to the normality test. According to the results, parametric tests for variables with normal distribution or nonparametric tests for variables without normal distribution were performed. The specific tests applied are indicated on the legend of the figures. A confidence interval (CI) of 0.95 and an alpha level of 0.05 were assumed, so a *P* value less than 0.05 was considered statistically significant. All the tests utilized were two-tailed. Statistical analyses were carried out using GraphPad Prism versions 5.00 and 7.03 (GraphPad Software, USA).

## 3. Results

### 3.1. Patient's Characterization

Demography characteristics of the subjects are demonstrated in Table 1. The breast cancer, benign breast disease, and control patients consisted of 10 women, each one with a mean age ± standard deviation (SD) of 53.3 ± 11.2, 41.5 ± 4.2, and 43.2 ± 16.0 years, respectively. The smoking and alcoholism patterns were similar (*P* > 0.05) in breast cancer, benign breast disease, and control patients. History of smoking had a frequency of 30% in breast cancer, 40% in benign, and 30% in control. Family history of breast cancer was reported only in cancer patients (40%). The clinical, hormonal, diagnostic, and therapy characteristics of patients with breast cancer are summarized in Table 2.

### 3.2. FTIR Analysis of Saliva Spectra between Breast Cancer, Benign, and Control Patients

**Figure 1:**
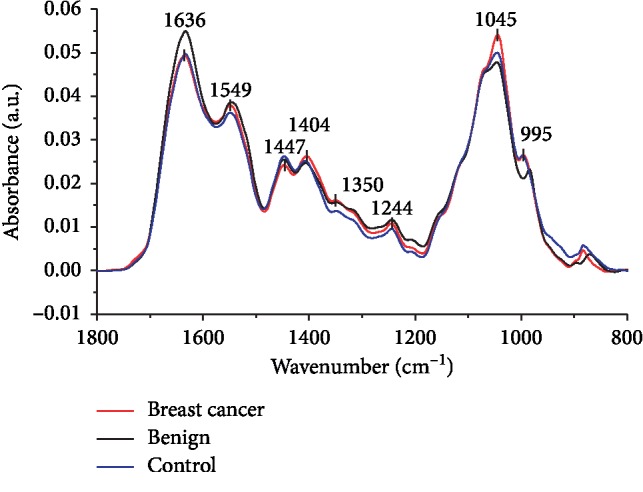
FTIR spectra for breast cancer, benign breast disease, and control saliva. Average original spectra with the absorbance bands of the major functional groups in biomolecules indicated between wavenumbers 1800 cm^−1^ and 800 cm^−1^ for breast cancer (red line), benign breast disease (black line), and control saliva (blue line).

The averages of the infrared original spectrum of whole saliva of breast cancer, benign, and control patients are represented in Figure 1 with a superposition of several salivary components as proteins, nucleic acids, lipids, and carbohydrates. The protein content is mainly attributed to wavenumbers at 1636 cm^−1^ and 1549 cm^−1^ that corresponds to amide I and amide II, respectively. CH_3_ asymmetric bending and *ν*_s_ (COO^−^) are related with wavenumbers 1447 cm^−1^ and 1404 cm^−1^, respectively. The wavenumbers 1350 cm^−1^ and 1244 cm^−1^ indicate amide III. The 1045 cm^−1^ and 995 cm^−1^ bands indicate *ν*_s_ (PO_2_^−^) and C-O ribose/C-C, respectively. A resume of the assignments of main vibrational modes and their respective salivary component is shown in Table 3.

**Figure 2:**
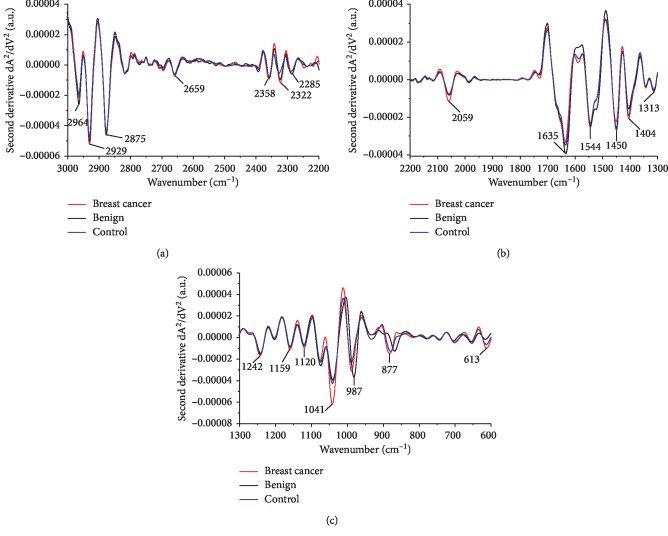
Detailed average second-derivative spectra and major wavenumbers. Average spectra between (a) 3000 cm^−1^ and 2200 cm^−1^, (b) 2200 cm^−1^ and 1300 cm^−1^, and (c) 1300 cm^−1^ and 600 cm^−1^ for breast cancer (red line), benign breast disease (black line), and control saliva (blue line).

The second-derivative infrared spectra of whole saliva of breast cancer, benign, and control patients were analyzed in detail to identify specific spectral components. The averages of the second-derivative infrared spectra of saliva for each group of patients are presented in Figure 2. The major wavenumbers detected in whole saliva were found at ∼2964, 2929, 2875, 2659, 2358, 2322, and 2285 (3000 cm^−1^—2200 cm^−1^ region, Figure 2(a)), 2059, 1635, 1544, 1450, 1404, and 1313 (2200 cm^−1^—1300 cm^−1^ region, Figure 2(b)), and 1242, 1159, 1120, 1041, 987, 877, and 613 cm^−1^ (1300 cm^−1^—600 cm^−1^ region, Figure 2(c)). The vibrational modes and related molecular sources of these wavenumbers are presented in Table 4.

### 3.3. Prevalidation as Diagnostic Potential by ROC Curve and Pearson Correlation

**Figure 3:**
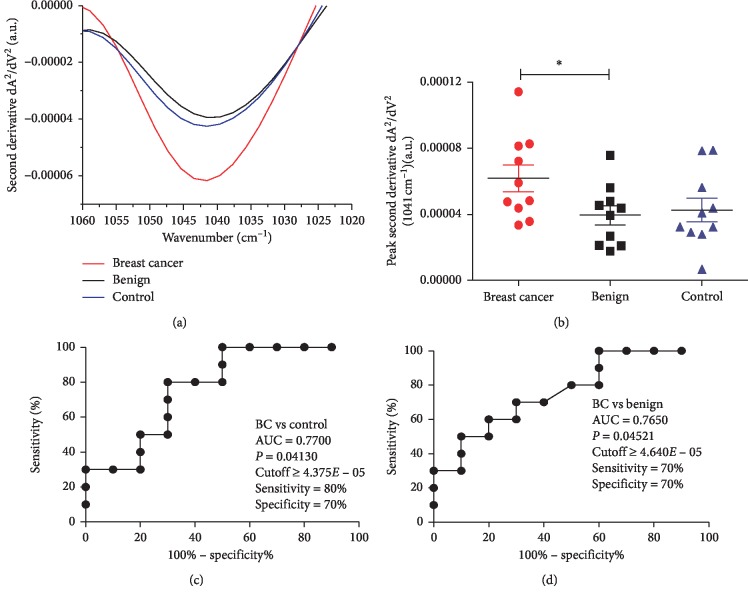
Comparison of the second-derivative absorbance of the statistically significant peak 1041 cm^−1^ between the three study groups. (a) Average second-derivative spectra between 1060–1020 cm^−1^ highlighting the wavenumber 1041 cm^−1^ for breast cancer (red line), benign breast disease (black line), and control saliva (blue line). (b) Scatter plot of the statistically significant wavenumber 1041 cm^−1^ for breast cancer (red), benign breast disease (black), and control saliva (blue). The line represents the mean, and the error bars (whiskers) represent the standard error of the mean (SEM) (^*∗*^*P* < 0.05, comparison of groups via the unpaired *t*-test with Welch's correction). ROC curves made from the wavenumber 1041 cm^−1^ for (c) breast cancer vs. control and (d) breast cancer vs. benign breast disease. Results about area under the curve (AUC), *P* value, cutoff, sensitivity, and specificity are being shown near the ROC curve. Statistically significant differences are represented by ^*∗*^ (^*∗*^*P* < 0.05).

Considering that sensitivity and specificity are basic characteristics to determine the accuracy of a diagnostic test, ROC analysis were used to ascertain the potential diagnosis of each vibrational modes of the original and second-derivative spectrum. A resume of statistical analysis (mean ± SD; *t*-test; ROC curve *P* value, sensitivity, and specificity) of all FTIR vibrational modes of the second-derivative spectra (described in Figure 2) are presented as supplementary material in [Supplementary-material supplementary-material-1]. Here, we show our results with more potential diagnosis between all bands analyzed, peak 1041 cm^−1^, and region between 1433 cm^−1^ and 1302.9 cm^−1^. The comparison of the 1041 cm^−1^ salivary vibrational mode in the second derivative of breast cancer, benign, and control patients is presented in Figure 3. This salivary vibrational mode was increased (*P* < 0.05) in breast cancer than in benign patients. However, this vibrational mode was similar (*P* > 0.05) in breast cancer patients and matched controls. Specifically, the vibrational mode showed higher absorption in breast cancer than in benign patients (*P*=0.039), and no matched significant difference compared with the controls (*P*=0.094). As expected, the 1041 cm^−1^ salivary vibrational mode was similar (*P*=0.740) in control and benign patients (Figure 3(a)). Since the 1041 cm^−1^ salivary vibrational mode can be used to discriminate breast cancer and benign patients, we evaluated the ROC curve and calculated the area under the curve (AUC) (Figures 3(b) and 3(c)). The ROC curve analysis shows a reasonable accuracy of ATR-FTIR tool to discriminate breast cancer from benign and control patients, with an AUC of 0.770 for breast cancer vs. control and an AUC of 0.765 for breast cancer vs. benign patients. Using the ROC curve, it was possible to select the optimal cutoff that distinguished breast cancer patients. This yielded a sensitivity of 80% and a specificity of 70% for breast cancer vs. control and a sensitivity of 70% and a specificity of 70% for breast cancer vs. benign patients.

**Figure 4:**
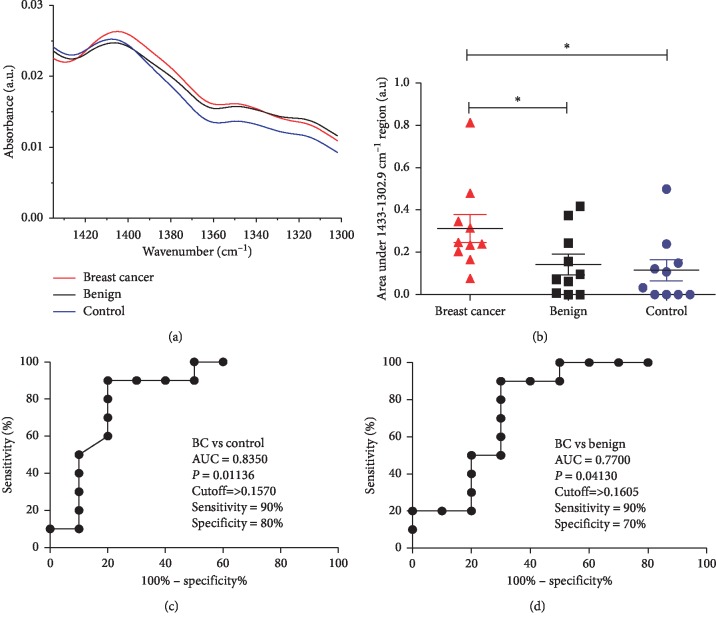
Comparison of the area under the 1433 cm^−1^ and 1302.9 cm^−1^ wavenumber region between the three study groups. (a) Average original spectra highlighting the 1433–1302.9 cm^−1^ region for breast cancer (red line), benign breast disease (black line), and control saliva (blue line). (b) Scatter plot of the 1433 cm^−1^ and 1302.9 cm^−1^ region for breast cancer (red), benign breast disease (black), and control saliva (blue). The line represents the mean, and the error bars (whiskers) represent the standard error of the mean (SEM) (^*∗*^*P* < 0.05, pairwise comparison of groups via the Mann–Whitney test). ROC curves made from area under the 1433 cm^−1^ and 1302.9 cm^−1^ region for (c) breast cancer vs. control and (d) breast cancer vs. benign breast disease. Results about area under the curve (AUC), *P* value, cutoff, sensitivity, and specificity are being shown near the ROC curve. Statistically significant differences are represented by ^*∗*^ (^*∗*^*P* < 0.05).

Considering the difference of the salivary original spectra in the region between 1433 cm^−1^ and 1302.9 cm^−1^, we performed quantitative analysis in breast cancer, benign, and control patients (Figure 4). The 1433–1302.9 cm^−1^ salivary wavenumber range was higher in breast cancer than in benign patients (*P*=0.0451) and matched control (*P*=0.0123) patients. It is important to note that the vibrational mode was similar in benign patients and control (*P*=0.5656) (Figure 4(a)). Since 1433–1302.9 salivary band area seems to be important for the discrimination of breast cancer from benign and control patients, we also evaluated the ROC curve between breast cancer and controls (Figure 4(b)) and between breast cancer and benign patients (Figure 4(c)). The ROC curve analysis shows a good accuracy of the ATR-FTIR tool to discriminate between breast cancer and the other groups of patients. The AUC of 1433–1302.9 salivary band area was 0.835 for breast cancer vs. control and 0.770 for breast cancer vs. benign patients. Using the ROC curve, it was possible to select the optimal cutoff that distinguished the groups of patients. This yielded a sensitivity of 90% and a specificity of 80% for breast cancer vs. control and a sensitivity of 90% and a specificity of 70% for breast cancer vs. benign patients.

## 4. Discussion

Our present data support our hypothesis that ATR-FTIR vibrational modes of saliva may discriminate breast cancer from benign and matched-control patients. Here, we have identified new salivary ATR-FTIR spectral biomarkers for breast cancer screening. The 1041 cm^−1^ salivary vibrational mode in the second-derivative spectra and the 1433–1302.9 cm^−1^ wavenumber region in the original spectra could potentially be used as salivary biomarkers to discriminate breast cancer from benign and matched-control patients with very good accuracy.

Our most potential spectral biomarker at 1433–1302.9 cm^−1^ was able to discriminate human BC from controls with sensitivity and specificity of 90% and 80%, respectively. Besides, it was able to differentiate BC from benign disease with sensitivity and specificity of 90% and 70%, respectively. Considering that mammography, ultrasound, and MRI, the conventional techniques used in clinical practice, show sensitivities of 67.8%, 83%, and 94.4% and specificities of 75%, 34%, and 26.4%, respectively [52], we believe that our results could improve the accuracy obtained for breast cancer diagnosis. However, in order to perform the conventional diagnosis, high-end equipments and facilities are required with significant clinical costs. Furthermore, circulating biomarkers have also been used as indicators of breast cancer; however, none of them has reached adequate sensitivity and specificity, limiting their clinical applicability in breast cancer diagnosis [53]. Infrared spectroscopy allows analyzing the entire biochemical signature (including proteins, lipids, nucleic acids, and carbohydrates) of a biological sample rather than focusing on a single specific protein as a biomarker [25]. Therefore, the salivary ATR-FTIR spectra are highly desirable due to their speed, convenience, and cost effectiveness, strongly suggesting this diagnostic platform for breast cancer screening.

ROC curve analysis is widely considered to be the most objective and statistically valid method for biomarker performance evaluation. In the current study, the ROC curve analysis showed reasonable accuracy for the salivary 1041 cm^−1^ level of second-derivative ATR-FTIR spectra and good accuracy for the 1433–1302.9 band area. The salivary 1041 cm^−1^ level of second-derivative ATR-FTIR spectra was increased in breast cancer patients compared with benign patients. Surprisingly, despite the absence of significant difference between breast cancer patients and controls, this spectral biomarker candidate exhibited significant diagnostic value with an AUC of 0.7700 comparing breast cancer patients than controls. Additionally, it also exhibited significant diagnostic value with similar AUC to compare breast cancer and benign patients. Therefore, this salivary spectral ATR-FTIR biomarker is a compatible complementary alternative to improve diagnosis of breast cancer. The 1433–1302.9 band area was elevated in saliva of breast cancer patients as compared with control and benign patients, and this band area showed a high sensitivity and specificity to discriminate breast cancer from both controls and benign patients, being prevalidated as a salivary ATR-FTIR biomarker of breast cancer by ROC curve analysis. The discriminatory power of this biomarker candidate for breast cancer reached 90% of specificity and 80% of sensitivity from matched controls and 90% of specificity and 70% of sensitivity from benign patients. As to potential for clinic application, these data strongly indicate that the salivary band area of the 1433–1302.9 cm^−1^ region had a high capacity do discriminate patients with breast cancer from healthy and benign patients. It is important to note that the salivary band area of the 1433–1302.9 cm^−1^ region was similar between benign and control, which is in concordance with blood test analysis [25].

It is known that increase in absorbance in each specific spectral vibrational mode represents increase in the presence of a specific biomolecule [44]. The increase in absorbance levels of breast cancer patients at the 1041 cm^−1^ vibrational mode is due to increased levels of PO_2_^−^ symmetric stretching [*ν*_s_ (PO_2_^−^)], which is present in nucleic acids and glycogen. Previous studies on cancer cells and tissues using FTIR spectroscopy also reported many changes in the phosphate region, which corresponds mainly to nucleic acids and carbohydrates [25]. The increased level in the 1433–1302.9 cm^−1^ region is due to increased levels of COO^−^ symmetric stretching [*ν*_s_ (COO^−^)], which is present in proteins and lipids.

Considering the higher expression of PO_2_ symmetric stretching (*ν*_s_ (PO_2_^−^)) and COO^−^ symmetric stretching (*ν*_s_ (COO^−^)) in saliva of breast cancer patients, we suggest that these molecules are originated from blood and access saliva by passive diffusion of lipophilic molecules (e.g., steroid hormones) or active transport of proteins via ligand-receptor binding [35]. Hence, saliva may present biomarkers that reflect the pathophysiological state of the body, such as, breast cancer. There are numerous putative salivary molecular biomarkers that are probably altered in the presence of breast cancer. Higher levels of some proteins [54–56], carbohydrates [52], and nucleic acids [47] have already been found in the saliva of breast cancer patients in comparison with normal controls, which corroborates with the results found in this study. In general, these biomarkers were evaluated by proteomic, immunological, and biomolecular techniques.

Higher levels of many proteins were observed in the saliva of breast cancer patients, such as (a) vascular endothelial growth factor (VEGF) and epidermal growth factor (EGF), which are potent angiogenic factors; (b) carcinoembryonic antigen (CEA) that is a glycoprotein and well-established serum tumor marker for breast cancer [54]; (c) soluble form of HER2 protein, that is a receptor tyrosine kinase, product of c-erbB-2 oncogene, and marker of poor prognosis [55]; and (d) p53 that is a tumor suppressor protein product of oncogene p53, it regulates target genes that induce cell cycle arrest, apoptosis, senescence, DNA repair, or changes in metabolism, and it is the indicator of poor clinical outcome [56].

One limitation of our study is the relatively small number of patients and the need for larger multicenter studies to confirm our results. Another limitation of this study is the lack of information about the specificity of this salivary ATR-FTIR spectral biomarker in breast cancer, especially considering that other cancers may also exhibit similar changes. Therefore, further studies are needed to evaluate the diagnostic performance of these spectral ATR-FTIR biomarkers of saliva in other cancers.

## 5. Conclusions

In conclusion, the present study showed for the first time that ATR-FTIR spectroscopy can be used in saliva samples to discriminate breast cancer patients than benign patients and healthy subjects. It was found absorbance levels significantly higher in saliva of breast cancer patients compared with benign patients at wavenumber 1041 cm^−1^ and the ROC curve analysis of this peak showed a reasonable accuracy to discriminate breast cancer from benign and control patients. In addition, we demonstrated that the 1433–1302.9 cm^−1^ wavenumber region was elevated in saliva of breast cancer patients as compared with control and benign patients. Our study highlighted this salivary spectral region as a biomarker with high accuracy to differentiate breast cancer from both control and benign patients. In summary, these innovative results suggest that salivary analysis by ATR-FTIR spectroscopy is a promising tool for breast cancer diagnosis.

## Figures and Tables

**Table 1:** Demography characteristics of breast cancer, benign breast disease, and control patients.

Characteristics	Breast cancer *n* = 10	Benign *n* = 10	Control *n* = 10
Age (years)			
Range	42.0–75.0	33.0–49.0	22.0–63.0
Average ± SD	53.3 ± 11.2	41.5 ± 4.2	43.2 ± 16.0
History of smoking (%)	30	40	30
Family history of breast cancer (%)	40	0	0

**Table 2:** Clinical, hormonal, diagnostic, and therapy characteristics of breast cancer patients.

Variable	Patients (*n* = 10)	
	*N*	%
Histological subtype		
Invasive ductal carcinoma	6	60
In situ ductal carcinoma	3	30
Mucinous carcinoma	1	10

Histological grade		
G2	5	50
G3	2	20
NR	3	30

Primary tumor		
ptx	1	10
pTis	3	30
pT1	4	40
pT2	2	20

Regional lymph nodes		
pNX	2	20
pN0	5	50
pN1	1	10
pN2	1	10
NR	1	10

Distant metastases		
pM0	7	70
NR	3	30

TNM staging		
0	2	20
I	1	10
II	2	20
NR	5	50

Status ER		
Positive	8	80
NR	2	20

Status PR		
Positive	8	80
NR	2	20

Status HER2		
Positive	2	20
Negative	6	60
NR	2	20

p53		
Positive	8	80
NR	2	20

Ki67		
≤14%	5	50
>14%	3	30
NR	2	20

Molecular phenotype		
Luminal A	4	40
Luminal B	4	40
NR	2	20

Therapy		
Surgery (S)	1	10
*S* + radiotherapy (RT)	1	10
*S* + RT + hormone therapy (HT)	3	30
*S* + RT + HT + chemotherapy (CT)	5	50

G1, grade 1; G2, grade 2; G3, grade 3; NR, not reported; ER, estrogen receptor; PR, progesterone receptor; HER2, human epidermal growth factor receptor 2; p53, tumor protein p53; ki67, antigen ki67.

**Table 3:** Assignments of main wavenumbers indicated in the average original saliva ATR-FTIR spectra of Figure 1 and assignments based on different references [45–48].

Peak (cm^−1^)	Proposed vibrational mode	Molecular source
1636	Amide I [*ν* (C=O), *ν* (C–N), *δ* (N–H)]	Protein
1549	Amide II [*ν* (N–H), *ν* (C–N)]	Protein
1447	CH_3_ asymmetric bending [*δ*_as_ (CH_3_)]	Protein (methyl groups)
1404	COO^−^ symmetric stretching [*ν*_s_ (COO^−^)]	Lipid (fatty acids)/Protein (amino acids)
1350	Amide III [*ν* (C–N)]	Protein
1244		
1045	C-O stretching, C-O bending of the C-OH groups [*ν* (C-O), *δ* (C-O)]	Carbohydrates (glycogen glucose, fructose)
995	C-O ribose/C-C; RNA uracil ring stretching	Nucleic acid (RNA)

*ν* = stretching vibrations, *δ* = bending vibrations, s = symmetric vibrations, as = asymmetric vibrations.

**Table 4:** Assignments of FTIR peaks of the average second-derivative spectra and assignments based on different references [45–51].

2^nd^ derivative peak (cm^−1^)	Proposed vibrational mode	Molecular source

2964	CH_3_ asymmetric stretching (*ν*_as_ (CH_3_))	Lipid
2929	CH_2_ asymmetric stretching (*ν*_as_ (CH_2_))	Nucleic acid/Lipid
2875	CH_3_ symmetric stretching (*ν*_s_ (CH_3_))	Lipid
2659	Unassigned band	
2358	O=C=O stretching	Carbon dioxide
2322	Unassigned band	
2285	N=C=O stretching	Nitrile
2059	C-N stretching of thiocyanate anions (SCN^−^)	Thiocyanate
1635	Amide I (*β*-sheet structure)	Protein
1544	Amide II	Protein
1450	CH_2_ symmetric bending (*δ*_s_ (CH_2_)) Methylene bending	Lipid and protein
1404	CH_3_ symmetric bending (*δ*_s_ (CH_3_))	Protein (methyl groups)
1313	Amide III	Protein
1242	Amide III PO_2_^−^ asymmetric stretching (*ν*_as_ (PO_2_^−^))	Protein Nucleic acid
1159	C-O stretching (*ν* (C-O)) CO–O–C asymmetric stretching (*ν*_as_ (CO-O-C))	Protein/CarbohydrateLipid
1120	Phosphorylated saccharide residue Mannose-6-phosphate	Carbohydrate Protein (glycoprotein)
1041	PO_2_^−^ symmetric stretching (*ν*_s_ (PO_2_^−^))	Nucleic acid (RNA/DNA) and glycogen
987	C=C bending	Monosaccharides and polysaccharides
877	*C*_3'_ *endo/anti A*-form helix	Nucleic acid
613	C-H out-of-plane bending	Cell membranes

*ν* = stretching vibrations, *δ* = bending vibrations, s = symmetric vibrations, as = asymmetric vibrations.

## Data Availability

The datasets generated and/or analyzed during the current study are available from the corresponding author on reasonable request.

## Supplementary Materials

Supplementary Materials The supplementary material file includes Table S1 that shows a resume of statistical analysis (mean ± SD; *t*-test; ROC curve *P* value, sensitivity, and specificity) of all FTIR peaks of the second-derivative spectra shown in Figure 2.
