# Supplementary material for: Attenuated Total Reflection-Fourier Transform Infrared (ATR-FTIR) Spectroscopy Analysis of Saliva for Breast Cancer Diagnosis
Source: J Oncol. 2020 Feb 10;2020:4343590. doi: 10.1155/2020/4343590 (PMC7035572; doi:10.1155/2020/4343590)
Supplement: Supplementary Materials — The supplementary material file includes Table S1 that shows a resume of statistical analysis (mean ± SD; t-test; ROC curve P value, sensitivity, and specificity) of all FTIR peaks of the second-derivative spectra shown in Figure 2. [file 4343590.f1.pdf]

Supplementary Material

Table S1. Statistical analysis of FTIR peaks of the average second derivative spectra. Abbreviations: BC = Breast Cancer, B = Benign, C = Control

2nd derivative peak (cm^{-1})	Breast Cancer (BC) (Mean±SD)	Benign (B) (Mean±SD)	Control (C) (Mean±SD)	t-test (P value)	ROC curve BC vs B (Specificity;Sensitivity; P value)	ROC curve BC vs C (Specificity;Sensitivity; P value)
2964	2.11E-06± 1.72E-06	2.59E-06± 1.96E-06	1.96E-06± 1.52E-06	>0.05[1]	80%; 50%; 0.5454	70%; 60%; 0.7055
2929	5.24E-06± 1.53E-06	5.09E-06± 1.50E-06	4.75E-06± 9.10E-07	>0.05[1]	70%; 60%; 0.5454	70%; 50%; 0.5454
2875	4.52E-06± 7.95E-07	4.61E-06± 1.04E-06	4.38E-06± 1.19E-06	>0.05[1]	60%; 60%; 0.7337	60%; 60%; 0.9097
2659	7.26E-07± 2.08E-07	6.48E-07± 2.11E-07	6.24E-07± 1.12E-07	>0.05[1]	60%; 60%; 0.3075	60%; 60%; 0.2265
2358	9.38E-07± 5.34E-07	8.41E-07± 5.69E-07	7.65E-07± 4.81E-07	>0.05[1]	60%; 50%; 0.6776	90%; 50%; 0.3644
2322	1.14E-06± 6.52E-07	9.48E-07± 8.60E-07	4.08E-07± 2.50E-07	0.007[2]	60%; 60%; 0.3447	90%; 80%; 0.0041
2285	5.93E-07± 5.22E-07	5.85E-07± 3.02E-07	3.83E-07± 1.82E-07	>0.05[1]	70%; 50%; 0.3075	60%; 50%; 0.5454
2059	1.16E-05± 1.29E-05	8.16E-06± 7.77E-06	7.66E-06± 8.17E-06	>0.05[1]	70%; 50%; 0.8501	70%; 60%; 0.4963
1635	3.34E-05± 1.16E-05	3.93E-05± 1.90E-05	3.49E-05± 1.21E-05	>0.05[1]	70%; 60%; 0.4727	70%; 70%; 0.5454
1544	2.56E-05± 9.86E-06	2.46E-05± 8.78E-06	2.33E-05± 1.01E-05	>0.05[1]	60%; 50%; 0.9699	60%; 30%; 0.8501
1450	2.24E-05± 9.94E-06	2.58E-05± 9.71E-06	2.67E-05± 9.80E-06	>0.05[1]	60%; 40%; 0.7913	80%; 50%; 0.4963
1404	2.06E-05± 7.31E-06	1.56E-05± 8.40E-06	1.91E-05± 1.05E-05	>0.05[1]	90%; 60%; 0.2124	70%; 70%; 0.2569

(Continued)

Table S1. Continued

2nd derivative peak (cm^{-1})	Breast Cancer (BC) (Mean±SD)	Benign (B) (Mean±SD)	Control (C) (Mean±SD)	t-test (P value)	ROC curve BC vs B (Specificity;Sensitivity; P value)	ROC curve BC vs C (Specificity;Sensitivity; P value)
1313	5.16E-06± 2.84E-06	6.25E-06± 3.10E-06	5.34E-06± 2.08E-06	>0.05[1]	80%; 60%; 0.4727	70%; 70%; 0.5454
1242	1.69E-05± 5.15E-06	1.47E-05± 5.84E-06	1.61E-05± 5.93E-06	>0.05[1]	80%; 70%; 0.2124	90%; 40%; 0.9397
1159	1.10E-05± 2.15E-06	8.74E-06± 3.83E-06	9.05E-06± 2.85E-06	>0.05[1]	80%; 60%; 0.1405	80%; 60%; 0.0822
1120	9.43E-06± 4.19E-06	7.54E-06± 9.35E-06	7.25E-06± 5.47E-06	>0.05[1]	60%; 50%; 0.9097	60%; 60%; 0.2569
1041	6.17E-05± 2.55E-05	3.95E-05± 1.83E-05	4.26E-05± 2.28E-05	0.0393[3]	70%; 70%; 0.0452	80%; 70%; 0.0413
987	3.13E-05± 2.04E-05	2.72E-05± 2.59E-05	2.32E-05± 1.53E-05	>0.05[1]	70%; 50%; 0.4727	70%; 50%; 0.4057
877	1.31E-05± 1.50E-05	5.12E-06± 3.87E-06	1.28E-05± 1.24E-05	>0.05[1]	70%; 50%; 0.3075	70%; 40%; 0.9397
613	9.88E-06± 9.76E-06	1.43E-06± 1.85E-06	6.40E-06± 7.66E-06	0.0032[3]	90%; 80%; 0.0046	90%; 60%; 0.2265

Note: [1] Represents p>0.05 to comparisons BC vs B, BC vs C and B vs C; [2] Represents p<0.05 to comparison BC vs C; [3] Represents p<0.05 to comparison between BC vs B.
